# From self-narration to a worldview: a phenomenological, narratological, and linguistic case study of a patient with a complex clinical picture of bipolar disorder

**DOI:** 10.3389/fpsyt.2025.1648141

**Published:** 2026-01-20

**Authors:** Aleš Oblak, Marko Vrbnjak, Alina Holnthaner, Nika Kovačič, Martin P. Kastelic, Tatjana Marvin Derganc, Jure Derganc, Vid Vanja Vodušek, Urban Kordeš, Jurij Bon, Borut Škodlar

**Affiliations:** 1 University Psychiatric Clinic Ljubljana, Ljubljana, Slovenia; 2 Center for Cognitive Science, Faculty of Arts, University of Ljubljana, Ljubljana, Slovenia; 3 Department of Comparative and General Linguistics, Faculty of Arts, University of Ljubljana, Ljubljana, Slovenia; 4 Institute of Biophysics, Faculty of Medicine, University of Ljubljana, Ljubljana, Slovenia; 5 Center for Cognitive Science, Faculty of Education, University of Ljubljana, Ljubljana, Slovenia; 6 Department of Psychiatry, Faculty of Medicine, University of Ljubljana, Ljubljana, Slovenia

**Keywords:** bipolar disorder, Jaspers, linguistics, narratology, phenomenological psychopathology

## Abstract

**Background:**

Comorbidity of bipolar disorder and aspects of neurodevelopmental disorders present unique challenges and opportunities in understanding the formation and maintenance of selfhood and worldview in psychopathology. Traditional cognitive models often overlook the narrative and phenomenological dimensions of patient experience, particularly how autobiographical narration and emotionally charged worldviews mediate lived experience.

**Objectives:**

This study aims to explore (i) how worldviews are shaped by emotion and memory in psychiatric illness, and (ii) how narrative forms provide existential coherence in psychopathology. The research adopts a multidisciplinary approach integrating psychiatric, phenomenological, linguistic, narratological, and hermeneutic perspectives, through an in-depth case study.

**Methods:**

A single-case study design was employed, focusing on “Benjamin,” a 60-year-old male with a lifelong history of bipolar disorder type I and suspected Asperger’s syndrome. Data sources included clinical interviews, autobiographical writings (five book-length texts), and clinical observation. Analyses were conducted using phenomenological, narratological, and linguistic frameworks to trace the evolution from self-narration to worldview construction.

**Results:**

Benjamin’s case illustrates a transition from immediate self-description to the development of a coherent, philosophy-like worldview. His autobiographical narratives reveal the interplay between minimal and narrative self, with mood episodes influencing both self-experience and identity coherence. Emotional memories—regardless of factual accuracy—serve as organizing phenomena, providing existential structure and meaning. The study highlights the limitations of cognitive schema theory and underscores the importance of narrative scaffolding and affect-logic in shaping worldviews.

**Conclusions:**

The study demonstrates how a multidisciplinary analysis of autobiographical narration can be useful for characterizing emotionally charged worldviews when working with individuals with complex, comorbid and chronic psychiatric disorders. Integrating phenomenological and narratological approaches yields a deeper understanding of selfhood and meaning-making in psychopathology, with implications for clinical assessment and intervention.

## 1 Introduction

Benjamin was walking through a field in the countryside. He had been troubled by a difficult family situation. His son had been the victim of bullying, which culminated in one of the parents formally asking the school for him to be removed from the class. In response, Benjamin found his son’s bully and physically beat him. In the aftermath, he experienced a sense of limited future. He found his life to be devoid of possibilities. He found an old rope in a barn and tried to hang himself. The rope broke. It might have been an act of God. It might have been that the rope was just old. Fate or circumstance? It never occurred to him to ask himself that question.

This moment crystallizes many of the themes that emerge across Benjamin’s existential concerns. He is a patient with bipolar disorder as well as several comorbid symptoms primarily related to social cognition and affectivity, which are indicative of a developmental disorder (most likely Asperger’s syndrome). Additionally, he has *situs inversus totalis*, a rare condition impacting about 1 in 10.000 births (1) where his internal organs are mirrored in structure and location. He is of interest due to the unusual development of his disorder and his pronounced interest in self-reflection, which is possibly rooted in his comorbidities. In attempting to understand and cope with his troubles, he produced five book-long texts in which we can see a clear transition from a description of his lived experience to a philosophy-like account of the world. His experience offers a valuable opportunity to investigate the relationship between lived experience, self-narration, and worldview, and their role in psychopathology.

In contemporary medicine, treating patients with multiple comorbidities has become routine, and psychiatry is no exception. Nevertheless, we must ask ourselves: is the coexistence of several psychiatric disorders truly analogous to having multiple somatic diseases? The concept of comorbidity usually assumes that each condition is a distinct, independent entity with its own etiology and pathology (2, 3). In psychiatry, however, these etiopathological foundations are often only partially understood. As a result, the boundaries between disorders are notoriously difficult to demarcate. Having elusive organic basis and unclear pathognomonic features, the major psychiatric classification systems, Diagnostic and Statistical Manual of Psychiatric Disorders (DSM) and International Classification of Diseases (ICD), rely solely on clinical descriptions. However, the diagnostic systems from DSM-III through DSM-5 have been criticized for their overly simplified categorical approach (4). What appears to be missing from current psychiatric diagnostic systems is an appreciation of a unifying phenomenological structure that gives coherence to individual symptoms. This perspective becomes especially important when we encounter patients with complex symptomatology and multiple diagnoses, as it can offer a deeper, more integrated understanding of their condition rather than reducing it to a collection of separate comorbidities.

One such organizing Gestalt is an unstable sense of self, which is common across a variety of psychopathologies (5). It includes altered body ownership and first-person perspective, resulting in disembodiment or depersonalization (6). Narrative instability may appear as fluctuating autobiographical memory and identity coherence (7). Key dimensions of self-instability are cognitive disruption, detachment, and blurred self-world boundaries (8), which may cause existential disorientation and functional impairment (6). In bipolar disorder, the sense of self is generally stable but can be transiently disrupted during mood swings: mania may cause hyper-reflexivity, while depression can reduce the sense of agency and bodily connectedness (9). In autism spectrum disorder, sensorimotor integration difficulties destabilize the minimal self, causing asynchrony between bodily actions and social stimuli (10, 11).

In bipolar disorder, the narrative self, shaped by autobiographical memory and social identity, is profoundly affected by mood fluctuations, leading to shifting and fragmented self-narratives (12). Difficulty distinguishing genuine self-aspects from mood-driven distortions complicates identity formation, while social stigma can reinforce fragmentation and isolation (13, 14). In autism spectrum disorders, communication challenges and external pathologizing narratives affect the narrative self, often leading to self-doubt and fragmented identity, though diagnosis can enable more accepting self-narratives (11).

Narratives we produce about ourselves are intentional and embedded in socio-historical contexts (7). Phenomenologically-oriented approaches focus on the embodied, lived experience as a source of one’s narrative self-understanding (15). On the other hand, psychological approaches take as their starting point a person’s underlying personality traits (16). Both approaches, however, stress that self-narratives are not fixed but evolve throughout our lives (16, 17). Once articulated, self-narratives become texts open to interpretation by others (18). Importantly, self-narratives can be fictional or symbolic, drawing on affectively charged memories that provide coherence to our existential understanding, regardless of their factual accuracy (7, 18). So-called *originary phenomena* [Ger. *Urphänomene*] (originally coined by Goethe) (19) or *self-defining memories* (20) are memories that we continuously bring to mind in order to make sense of our broader existential situation. The events recounted in these memories need not have actually happened. It is through their affective charge, vividness, and dense interconnectedness with other memories, not their veridicality, that we make sense of our lives (20).

Constructing coherent narratives is particularly important when we experience so-called free-floating or unattached feelings. They do not pertain to a specific affective stimulus, but appear unprompted in one’s consciousness (17). These are linked to the so-called “growth of private worlds”. They are further associated with feelings of insight and bliss, and pertain to the most profound aspects of life, such as timelessness, the divine, and mortality. These are particularly relevant in limit situations [Ger. *Grenzsituationen*], where existential crises (typically difficult but unavoidable experiences such as bereavement and coping with one’s mortality) disrupt our psychological functioning. In these states, worldviews offer us both existential safety (e.g., using religion to cope with loss) and are themselves subject to change and growth (e.g., taking up a religious worldview after having a near-death experience) (17, 21, 22). In limit situations states, people might abandon old systems of morality (e.g., such as Benjamin physically beating his son’s bully).

Jaspers (17) describes worldviews as comprising a world-picture [Ger. *Weltbild*] and personal attitudes [Ger. *Einstellungen*] toward it. Together, they form an enclosure [Ger. *Gehäuse*], which provides coherence to our lived experience and shields us from the stressors of life. Worldviews must emerge organically from lived experience rather from received frameworks (23). Crucially, worldviews need not comport with factual reality. Bortolotti (24) speaks of so-called epistemic need (the need to know) wherein delusional beliefs are endorsed because they accommodate a person’s lived experience, rather than being epistemically sound.

Relatedly, autobiographical recollections are not neutral, factual accounts of the past, but are continuously (re)constructed (25, 26). This process involves individuals reflexively evaluating their experiences, weighing them against personal, moral and existential values. This dynamic narrative process aligns with Depraz (27), who emphasizes that the evocation of specific, prereflective experiences often triggers associative chains that evoke affectively charged memories, influenced by one’s current stance toward their personal history.

In linguistics, there is a fundamental idea that when we produce communicative acts we necessarily do so by referencing events in our lifeworld (28, 29). This allows a humanistic investigation of how a person’s lived experience shapes their self-understanding. This practice is commonly done with eminent personages. Aby Warburg was a humanistic scholar whose psychoses cantered around snakes, which, in turn, contributed to his academic interest in serpent rituals (30). Ludwig Wittgenstein’s logical atomism may have been related to his persistent sense of loneliness and isolation (31). While the translation of lived experience into narrative is a universal aspect of human communication, Benjamin’s specific condition affords us the opportunity to investigate how this process plays out in a psychiatric patient with a complex clinical picture. It allows us to see how sensemaking may contribute to coping with and perhaps the perseverance of psychiatric symptoms.

Thus, the focus on worldview and narrative highlights how individuals continuously construct meaning and identity through emotional experience and storytelling, navigating the interplay between their inner life and the external world. This framework emphasizes the dynamic, interpretive nature of selfhood and the protective, structuring role of worldviews in human experience. In the context of this study, we do not refer to worldviews in terms of a set of propositional beliefs, but to a pre-reflective structure of meaning that shapes one’s sense of coherent world (17, 32). While contemporary cognitive science often emphasizes experience as it is present in the moment, due to concerns over memory’s reconstructive nature (33–37), the importance of autobiographical perspective remains. Jaspers (17, p. 671) pointed out that in a biographical study [Ger. *Biographik*] the factual sequence of events is less important than the “qualitative shaping of the living elements into temporal form?.

In response to these complexities, this paper adopts a multidisciplinary approach, using a detailed case study to illustrate how various perspectives (psychiatric, phenomenological, linguistic, narratological, and hermeneutic) can deepen our understanding of how worldviews are formed and maintained in psychopathology. This paper investigates how autobiographical narration and emotionally charged worldview-construction mediate the lived experience of a complex, comorbid and chronic psychiatric disorder. Our goal is to illustrate in a non-reductionistic manner how phenomenological, narratological, and linguistic methods can be used to investigate the construction of a specific worldview on the basis of lived experience of psychopathology. Through the case of Benjamin, we ask: (i) How are worldviews shaped by emotion and memory in psychiatric illness? and (ii) How do narrative forms provide existential coherence in psychopathology?

## 2 Method

### 2.1 The case

#### 2.1.1 Clinical, developmental, and social history

The case, anonymized as Benjamin, is a 60-year-old man. He has a lifelong history of mental health issues. His current diagnosis is bipolar disorder type I. He has experienced five major episodes of mania, each followed by depression, and has been hospitalized during each of these episodes. During depression, Benjamin’s chief complaint is fatigue. During mania, he exhibits flight of ideas, of which he is insightful but not critical. Benjamin has been diagnosed with *situs inversus totalis* and attempted suicide once. His manic episodes have been treated with Lithium or other mood stabilisers in the past, but his preferred stabilization treatment remains Olanzapine 5mg daily.

Benjamin exhibits difficulties with social cognition and emotions. He has difficulties with both cognitive and affective empathy. While emotional reactivity and prosocial behavior tend to oscillate in bipolar disorder (e.g., in hypomanic states, patients may exhibit at least apparent heightening of social skills), Benjamin presents with an atypical development of his disorder, where his emotions and behavior in interpersonal situations do not vary. This has led to differential diagnostic (DDX) consideration of a comorbid developmental or personality disorder. Asperger’s syndrome was considered, especially since his son was formally diagnosed with this disorder, which has a strong genetic component (38). However, since Benjamin’s possible autistic traits were undiscovered until middle adulthood, this determination could not be made for certain. We thus employed a strategy of elimination. Schizotypal personality disorder was considered; however, it was rejected by a clinical psychologist. Thus, we can claim within reason that Benjamin exhibits Asperger-like difficulties in social cognition (primarily mentalizing) and affectivity (primarily emotional reactivity).

As a child, Benjamin had issues with eye contact but learned to maintain it on his father’s insistence. He maintains five close friendships, has a wife and two children. He has an undergraduate degree in electrical engineering, a master’s degree in business administration, and an associate’s degree in theology. He is deeply religious and prays every night but does not attend church.

#### 2.1.2 Everyday functioning

Benjamin finds others difficult to understand. He works as a software engineer at a manufacturing company albeit with a reduced workload. He has several special interests (model planes, radio equipment), he is a picky eater (dislikes fruits and textured foods; his favorite dish is pancakes). He has difficulties with social formalisms (e.g., Slovenian exhibits T-V distinction and in both the medical and research settings, he consistently employs the informal mode). He was polite during the interviews. He has difficulties remembering names (e.g., he forgot the names of the principal investigator (PI), his psychiatrist, and clinical psychologists, despite prolonged contact). His mood is variable, ranging from dysthymic to hyperthymic, which is detectable by the researchers, but he is not always insightful to this.

### 2.2 Data collection

We drew on two sources of qualitative material. The main method of data collection were phenomenological interviews. The interviews were conducted over an eight-month period during a predominantly euthymic period (with occasional bouts of dysthymia). The interviews were conducted in-person and audio recordings were made.

The interviews were based on the method of lifeworld analysis (18, 39). Lifeworld analysis attempts to describe a patient’s phenomenological Gestalt by drawing on their lived experience of body, time, place, embodiment, others, emotions, and values. It assumes a researcher who actively attempts to empathize with the patient by using specific questions to attune to their experience. Rather than presupposing objective data, the qualitative material is thought to be hermeneutical in the sense that a) it amounts to the patient’s interpretation of their symptoms, and b) the researchers further interpret them. In total 19 interviews were conducted by a researcher with several years of experience with descriptive psychopathological interviews [AO]. Three debriefing sessions were conducted to ascertain Benjamin’s perspective on the research process. Additionally, the Examination of Anomalous Self-Experience (EASE) (8) semi-structured interview was conducted by a clinician licensed in this method [AH].

The second source of qualitative material was provided to us by Benjamin himself. He keeps a detailed record of various parts of his life (e.g., books he has read, films he has watched, model planes he bought), which he stores in a large Excel spreadsheet. Over the years, he produced five book-length texts reflecting on his experience.

### 2.3 Analysis

#### 2.3.1 Phenomenological analysis

The interviews were transcribed verbatim. The transcripts were analyzed within the framework of constructivist grounded theory (40). Grounded theory employs parallel analysis, where the data collection and analysis processes occur simultaneously. Our main analytical tool was coding. As the analysis yielded new understanding of Benjamin’s lifeworld, this informed our further data collection process. In subsequent sessions, we explicitly asked Benjamin about whether the emergent codes resonate with his experience.

Several forms of coding were employed. Sections of raw text were first analyzed inductively (i.e., they were grouped together based on their descriptive and conceptual similarity without reference to pre-existing theoretical frameworks). Second, the data were analyzed in an inductive-deductive manner. In the final stage, deductive coding was used (i.e., the final taxonomy of categories was fitted onto the data). These three analytic strands were treated as parallel but mutually informing approaches, allowing for theoretical triangulation between subjective experience, narrative construction, and language structure. The data collection and analyses processes were spearheaded by a researcher with a decade of experience with qualitative research who is certified in several interviewing and analyzing techniques [AO]. Coding was conducted independently by two researchers [AO & AH].

Constructivist grounded theory (40) assumes a constructivist epistemology; that is, it treats knowledge not as a recovery of objective, pregiven reality, but as a socially situated and contextually sensitive negotiation of meaning (41). However, it bears stating that while our understanding of Benjamin’s lifeworld is constructed, this was not done arbitrarily. We periodically organized meetings of a conciliary team including a psychiatry resident [AH], senior psychiatrists [JB & BŠ], including Benjamin’s attending physician [MV] and his clinical psychologist [VVV]. In these meetings, the emergent categories were critically discussed and revised until the whole team agreed on the interpretation that is a) theoretically sound; and b) accommodates Benjamin’s lived experience. EASE was analyzed according to the relevant guidelines (8) by two researchers formally trained and certified in this method [AH & BŠ].

As a means of both final validation and patient empowerment, Benjamin was shown an early draft of this paper. He was further asked about whether: a) he obtained any new self-knowledge from the project; and b) he has a sense of being understood by the research team. He reported positive changes in both.

#### 2.3.2 Narratological analysis

To investigate the narrative structure in Benjamin’s autobiographical writings, we conducted an in-depth narratological analysis. Specifically, we analyzed three of his most representative self-reflective texts: *Being—Theory*, *Being—Confession*, and *Grabbing and Absolution*. These texts were selected for their autobiographical depth and the insight they offer into Benjamin’s lived experience.

The narratological analysis proceeded in explicitly defined steps. First, each text was read holistically to identify dominant narrative configurations. Second, we coded narrative voice in terms of person (homodiegetic vs. heterodiegetic) and focalization (restriction of narrative information at the level of the story), following Genette’s (42) and Cohn’s (43) typologies. Third, we analyzed temporal structure by mapping the ordering of events (e.g., chronological progression, analepses and prolepses), the relative duration and pacing of narrated scenes, and the frequency with which key events or periods are retold. Fourth, we examined degrees of narrating distance, operationalized as the balance between experiential immediacy (scene-like, present-tense descriptions by the experiencing self) and retrospective, evaluative commentary by the narrating self. Finally, we assessed coherence of narrative identity as the extent to which the narrator offers a stable self-position across the text (consistency of self-descriptions, recurring life themes, and explicit attempts to integrate discrepant episodes into a meaningful whole), drawing on Ricœur’s notion of narrative identity and on phenomenological psychopathology (44, 45). These steps allowed us to compare the three texts not only qualitatively but also along shared narrative dimensions, and to offer a procedure that can be applied in future case studies.

#### 2.3.3 Linguistic analysis

Linguistic analysis of the texts employed CLASSLA-Stanza (46, 47), a recently developed tool for automated text analysis of South Slavic languages. CLASSLA-Stanza is built upon the Universal Dependencies (UD) framework (48), a globally coordinated annotation scheme that enables cross-linguistic comparison through consistent morphosyntactic annotation and which has already been applied to corpora in over 130 languages worldwide.

Text T1 (*Being—Theory*, 35,989 words) was written during the euthymic stage, Text T2 (*Being—Confession*, 64,434 words) was composed during depressive stage, and Text T3 (*Grabbing and Absolution*, 46,676 words) was produced during a manic episode. First, all non-narrative elements were removed from the original text along with the passages in languages other than Slovenian, and the text was segmented into individual sentences. The remaining Slovenian narrative content was processed using CLASSLA-Stanza on a local workstation. This tool performs part-of-speech (POS) tagging (verb, noun, adjective, etc.) and annotates the text with UD relations, which capture the grammatical relationships between words within sentences. Finally, quantitative language analysis was performed on the tagged and annotated text using custom written Python scripts.

The linguistic analysis compares the three texts in terms of both semantic and syntactic features. The semantic analysis specifically focuses on lexical diversity and vocabulary size, employing a well-established metric: the Type-Token Ratio (TTR) (49). In this context, tokens refer to the total number of words in the text, while types denote the number of unique word forms.

The TTR is calculated using a 500-word window that moves through the entire text (50). The standard measure of lexical diversity is the mean value of the TTR over the entire text, however, we also calculated the standard deviation of TTR to address its coherence. We assessed the syntactic complexity of the three texts using the following quantitative measures, applied to CLASSLA-Stanza–annotated texts: part-of-speech distribution, sentence length, and clause density. Part-of-speech distribution refers to the relative frequencies of different word classes. Additionally, we also quantified sentence length (defined as the average number of words per sentence) and clause density (the number of clauses per sentence).

The linguistic analysis was not undertaken in isolation but used to support interpretive claims from the phenomenological and narratological readings, especially regarding lexical variation across mood states and coherence of narrative identity.

## 3 Results

### 3.1 Phenomenological findings I: self and affectivity

On EASE, Benjamin scored positively on only 10 items out of 57, including obsessive symptoms, inability to split attention, hyperreflectivity, somatic depersonalization and magical thinking, which all can occur across different diagnostic categories and are not exclusive of schizophrenia spectrum disorders (51). When asked to describe his thoughts and embodiment, these were described as negations (e.g., “I have no problems concentrating,” “My body is okay. It functions. It is as if it is not there.”).

Benjamin has *situs inversus totalis.* For him, his right-heartedness is a symbol both of him being inherently unusual, and consistently disregarded by his surroundings^1^. Imagine, if you will, Benjamin in his childhood room with his sister. He was excited to learn that, unlike hers, his heartbeat was more readily felt on the right side of his body. She welcomed this discovery with a series of slurs. Later, medical professionals commonly reacted to him informing them of his right-heartedness with disbelief.

For Benjamin, the primary experience of the body is that of a body-as-an-object. The experiential distance further applies to his flattened affect and alexithymia. He recognizes dysphoria in fatigue, and mania in racing thoughts. Benjamin’s body detachment may echo early childhood experiences. He describes the memories of a tyrannical father, who treated him as a servant. He referred to him as his “wife’s son,” and made him feel “like an object.” His father was a domineering figure, with all other family members being merely tools at his disposal: “I feel that my father always moved against us. He used us to create an empire of his own”.

Benjamin has consistently reported feeling upset by others attaching simple descriptive tags onto him. His father labelled him a “hacker” for his technical knowledge. He is upset when others consider him “ambitious” and “materialistic.” Others projecting these simplified roles onto him reminds Benjamin of existing for others. He is himself ambivalent about this relationship. On the one hand, he consistently remarked on his need to feel useful: “I think it is my duty to help my children to live a good life.” This extends to strangers as well. When, during a trip to the United States, he met a woman who was planning a trip to Slovenia, he offered to prepare an overview of sights to see. Furthermore, he tends to describe others by quantifying them (e.g., how many degrees they have, their salaries, and how large their apartments are). Herein lies a paradox of Benjamin’s experience: he is upset when objectified by others and at the same time consistently does this to himself and others.

His self-objectification was perhaps most clearly seen in one interview where Benjamin shared an old photograph of him and his father working on plastering a wall. He asked the researcher whether he could see the “malice” in his father’s face. Then, he pointed to himself as a boy. Referring to himself in the third person, he asked: “If you look at this facial expression, can you see that he is afraid? Can you see that something is unnatural there?” He then added with urgency in his voice: “Do you have this feeling or don’t you?

Benjamin experiences a base level of social anxiety. Although he has a close-knit group of friends, who he is regularly in contact with, he reports being uncomfortable with strangers. When attending dancing lessons with his wife, he is relieved when they can leave. While none of us *know* for certain that people who we are interacting with like us, we make a Kierkegaardian leap of faith (52), trusting our gut feeling that we will know whether a social interaction is going awry. Benjamin is incapable of such a leap of faith. Thus, approximately once per interview session, he asked whether his story was interesting to us, whether it was useful, and remarked that he hopes he is not wasting our time.

In addition to these more grounded affective and interpersonal difficulties, Benjamin also frequently reports experiences of synchronicity. For example, during his participation in the study, he was attending a psychosocial support group. There, another patient described being yelled at “like a five-year old.” This was the same turn of phrase that Benjamin’s friend used to describe his wife’s attitude towards him. Benjamin mentioned this event several times during the interviews. He interprets such synchronicities as meaningful, causal coincidences that affirm his sense of uniqueness. They operate as moments of validation and subtly connect his internal life to a broader metaphysical order.

### 3.2 Phenomenological findings II: time and agency

Benjamin exists in an unusually broad timeframe. He feels as if the distant past (e.g., the life of his grandfather whom he never knew) affects the present, and that, in turn, his present actions echo in eternity. This broad time frame creates a fear of indefiniteness in Benjamin. For example, he feels uneasy when his projects are unfinished. The broad time frame that Benjamin inhabits creates a particular intellectual problem for him. *How to make sense of his existence?* We can see Benjamin’s experience of a broad timeframe reflected in the books he has written about himself. The interpretative framework through which he attempts to understand his life includes psychoanalysis, theology, electrical engineering. He writes about communism, inheritance laws^2^, genetics, and clinical psychology^3^.

To feel in control of our actions, we need to be able to observe or at least conceptualize their consequences (53). The potentially infinite duration of the consequences of his actions fosters a marked attitude towards agency in Benjamin. He does not feel helpless. In fact, he feels powerful, being able to “dominate over people” (citing examples from court proceedings and his military service where he, against all odds, came out on top in interpersonal conflicts). However, he is afraid of his own agency. There was a particularly salient episode when this fear was realized. His son had been bullied in school. He and his wife considered signing him up for a different class. Benjamin refused:

Whatever you do, you cause a massive change, and it creates responsibility. We actually do not know how it would have played out. Was this a bad path? [ … ] Maybe if we had transferred him to another school, the mess would have been bigger? It would have erupted later on. Maybe it would have been different and more devastating.

Furthermore, the presence of the-future-in-the-present makes Benjamin emotionally vulnerable. Due to the difficult relationship with his parents, he did not attend his father’s funeral and plans to do the same with his mother. The prospect of her death is something that he thinks about daily. He anticipates emotional turmoil associated with this event and aims to “harden” himself against it.

A further example of the relationship between lived time and psychological distress for Benjamin is his suicide attempt. The lived experience that grounded the suicidal impulse was what Benjamin referred to as “life no longer being promising^4^.” Following the bullying that his son experienced in school and Benjamin assaulting his classmate, he was prescribed antipsychotic and antidepressant medication. Benjamin suddenly experienced a collapse of the lived future. His experience of temporality, usually so extensive, was suddenly limited to the present moment. Everything seemed determined and his appraisal of the situation was fatalistic:

I thought about it in terms of everything being over. The only sentence that was present in my mind was that I cannot live like this. [ … ] They wouldn’t allow me to live any other way.

He felt “constricted” and “without a way out.” Fearing that his change in experience was due to psychiatric medication, he noted: “I read that it ruins you forever.

The close relationship between the experience of time and agency can be tied to Benjamin’s faith. He nominally completed his catechesis before marrying his wife, but became religious in earnest after an in-patient at the clinic gifted him *On Grace and Free Will* by St. Augustine.

### 3.3 Narratological findings

Benjamin’s insights exhibit consistent phenomenological structure. In manic states, Benjamin describes insights into the nature of himself and the world. These are typically rendered as simple tropes (e.g., *people should pay for their sins while they are alive*, and *the person who inherits the home takes care of their parents*^5^). Furthermore, these insights often reflect his technicist understanding of the world. He compares his mind to a neural network feeding on incoming data, interpersonal relations to the optical refraction of light and the Fourier transformation of a time series into the frequency domain, and his emotional stability to electrostatic forces.

Since early childhood, Benjamin has been wondering whether he is normal. This prompted him to try and learn more about his family’s past. Eventually, he found diaries and letters outlining some of the family history (e.g., his father being a member of the Yugoslav secret police). The archival documents created clarity for him, offering a lens through which he could understand why he felt different all his life. Family archives may have fueled Benjamin’s drive for self-reflection and meticulous record-keeping. Benjamin wrote five autobiographical novels as he calls them:

- *Being—Confession* (2013): his attempt at self-understanding;
- *Being—Theory* (2013): his worldview derived from his lived experience;
- *The Rules of Inheritance* (2015)*;*
- *Grabbing and Absolution* (2018): consisting of momentary descriptions of his lived experience as it is unfolding during a manic phase of his bipolar disorder;
- Dedications (2023).

*The Rules of Inheritance* consists of archival documents surrounding the issue of inheriting his father’s property. *Dedications* consist of photocopied dedications others have left for him in books and postcards. Benjamin supplemented the file with the comment: “*These also say a lot*.” Nevertheless, as these two documents are predominantly not autobiographical in nature, they are less relevant for our purposes and will not be analyzed.

*Being—Confession* and *Being—Theory* appear as relatively homogenous texts. The first is an experiential account of Benjamin’s life, reflecting his personal story. The second offers a distanced perspective shaped by his worldview. Narratological analysis revealed three main thematic domains: *narrative distance, causal coherence vs. fragmentation*, and temporal logic.

More closely, we can see *Being—Confession* as a chain of Benjamin’s life events, described with exaggerated or inadequate narrating distance (15). Benjamin describes them as if belonging to someone else. The text emphasizes the experiencing self over the narrating self with moments of self-reflection and thus of narrative identity occurring but briefly:

*Things moved from bad to worse. Father drank more. He raved more. We had to jump around him. I recall the drives between the vacation house and the apartment. There was an unbearable tension in the car. Father yelled at mom. When my sister and I were younger, we cried, but now that is gone. At night, in the apartment, in the bedroom, he often spent a long time yelling at mom. From this period, I recall that all of my sister’s boyfriends came from an altitude of above 800 meters. Even today, she is married at that altitude. The air must be good there. I wanted to say that the usual trend is to move from the hills into the valley (towards a better life and not the other way around). [ … ] Father was not at all disturbed by this. However, when she started dating a manual laborer and went out dancing every night, mom took notice. She found out that the boy has epilepsy and that was the criterion that ended the relationship. Mom did it in a fairly rough way. One has to wonder whether a person has the right to do that?*

*Being—Theory* describes Benjamin’s “insights,” which clarified his otherwise un-understandable^6^ world. These epiphanies do not follow any perceptible system or thread line. The text appears incoherent. It lacks transparent argumentation and associatively jumps from one topic to another. Consider one entry, titled How to Read a Person II (more elaborated):

*We do it by asking what others think of us. I happened to find out that I do this without wanting to. That is to say, subconsciously. It is like a “ping” or a sonar. I bit my tongue, explained the background of this question, and apologized. The topic just so happened to go to a very personal level, so I asked him what time it is.*

In some instances, his insights are grounded in explicit experiential episodes. However, on most occasions the source of insight is not transparent. Another characteristic is the fluidity of levels of abstraction and arbitrariness of Benjamin’s insights (54). In an entry called *Body*, he writes:

*I was very surprised. I am attending lectures (workshops) guided by a wonderful and very educated man (a priest, PhD, PhD, PhD). I expected that we will talk about God, soul, and similar such spiritual matters. [ … ] I was surprised by how much emphasis was placed on the body. The body never lies. You fell in love with the body … I was not counting it, but I feel that he (that is, the priest) is emphasizing the body. Father read about socialist morality and ethics (that is, he was a communist). Interesting. I thought that there is only one ethics and morality (as a reference). Maybe you have to say out loud according to which one you are behaving. A lot of things are different than one would expect.*

In sum, Benjamin’s worldview (17) in *Being—Theory* is primarily composed of technical, psychological and theological attitudes, scattered world-images and intense emotion-driven dynamism. Consider the entry titled Original Sin:

*I will use mathematical notations. That is how I discovered this. Our present moment is impacted by our entire history from less-than-infinite to now. Of course not all of the past influences us to the same degree. You can say that the near past is more impactful, the far past less. The same applies for space. We can express this as a summation (an integral of all the contributions from less-than-infinite to now. Of course, this begs the question of how to weigh individual actions. This does not really influence the model, but the realization. We can break this down into two parts. On the integral from the zero point and the second moment onward. The zero point is, of course, birth. The first part is called the original sin. The second part is our own sins. It can also have a plus or a minus sign (if our good acts are greater than our sins). Let us test this model in practice.*

The third text *Grabbing and Absolution* was written during a manic episode. It is much more fragmented and filled with letters, mails, descriptions of meetings and other fragments. Moments of self-reflection are extremely rare. We can assume that the emotional storm of manic episodes additionally destabilized the patient’s capacity to (self-)reflect, leaving him no other anchor than factual and concrete descriptions of events. Consider the following entry reflecting on a meeting about inheritance. The difficult emotions he feels towards his family mix with his religious considerations, his technicist understanding of interpersonal relationships and his worldview:

*For four hours, nobody had visited father and his mouth was dry (like Christ). Before that, four servants served him. Would it not be better if it were only one? Is it better that one job is done by one person or should they work in shifts? She said that he was locked up so that nobody could steal anything. Clearly! (Property is essential). They had the key. Instead of saying that somebody should always be next to a dying person, especially with inheritance like that, it would be appropriate for it to be taken care of. But they went 1/x. There were four of them so that nobody could steal anything. [ … ] I don’t even know what happened at night.*

### 3.4 Linguistic findings

**Figure 1:**
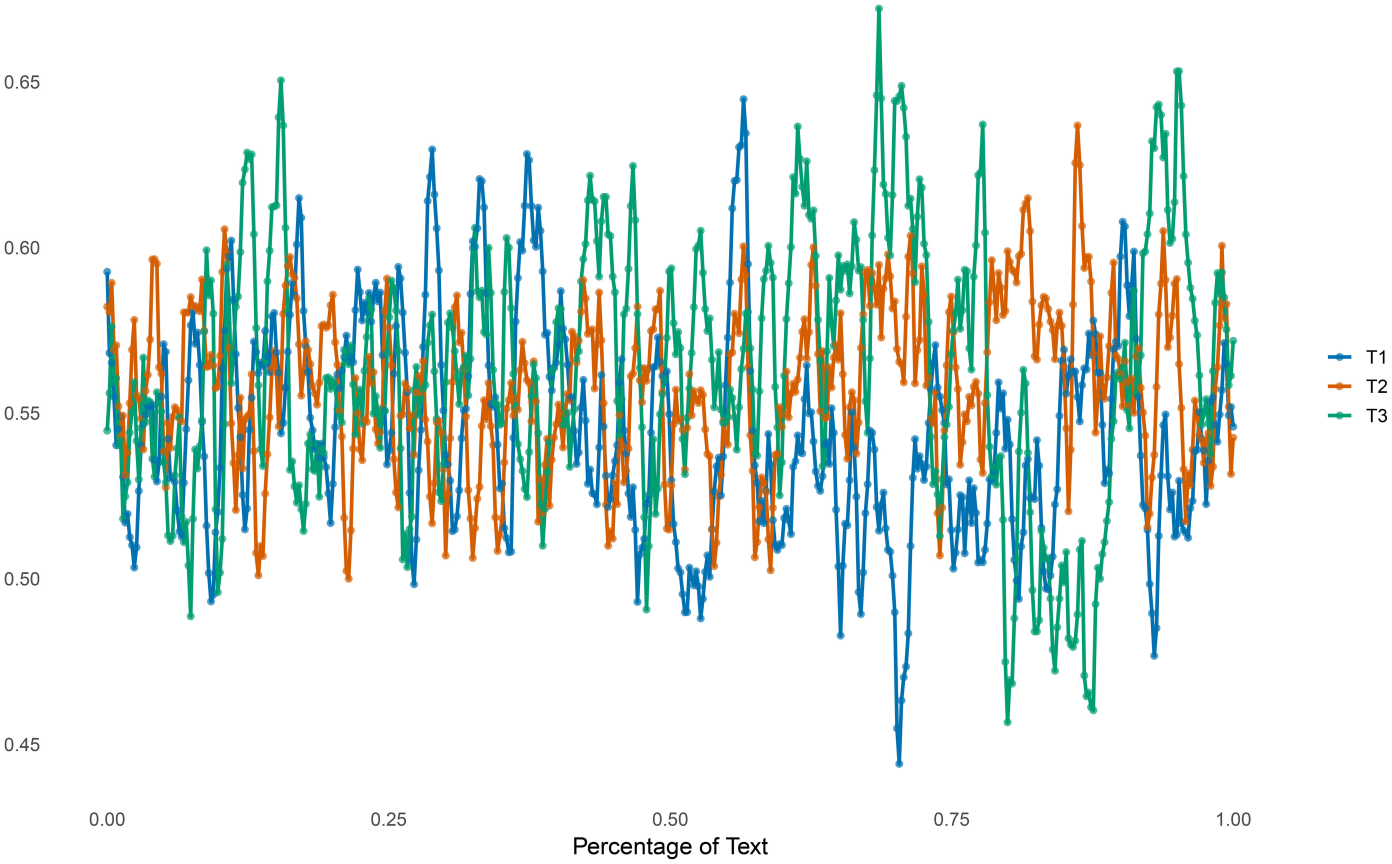
Changes in the type-token ratio (TTR) through T1, T2, and T3.

The mean TTR values across the three texts are relatively similar; however, their standard deviations differ notably (**Table 1**). T2 exhibits the lowest standard deviation, while T1 shows a 30% increase in comparison. T3, produced during the manic episode, has the highest standard deviation—21% higher than T1 and 60% higher than T2 (see **Figure 1**). These findings suggest that the patient’s overall vocabulary size remains relatively stable across the T1 and T2 phases as well as during the manic episode. However, the markedly higher variability in the manic phase points to increased inconsistency in lexical use during that period.

**Table 1:** Standard deviation of TTR in Texts T1, T2 and T3.

Measures of TRR	Text T1	Text T2	Text T3
Mean TTR:	0.545	0.559	0.563
SD TTR:	0.0329	0.0250	0.0399

**Figure 2:**
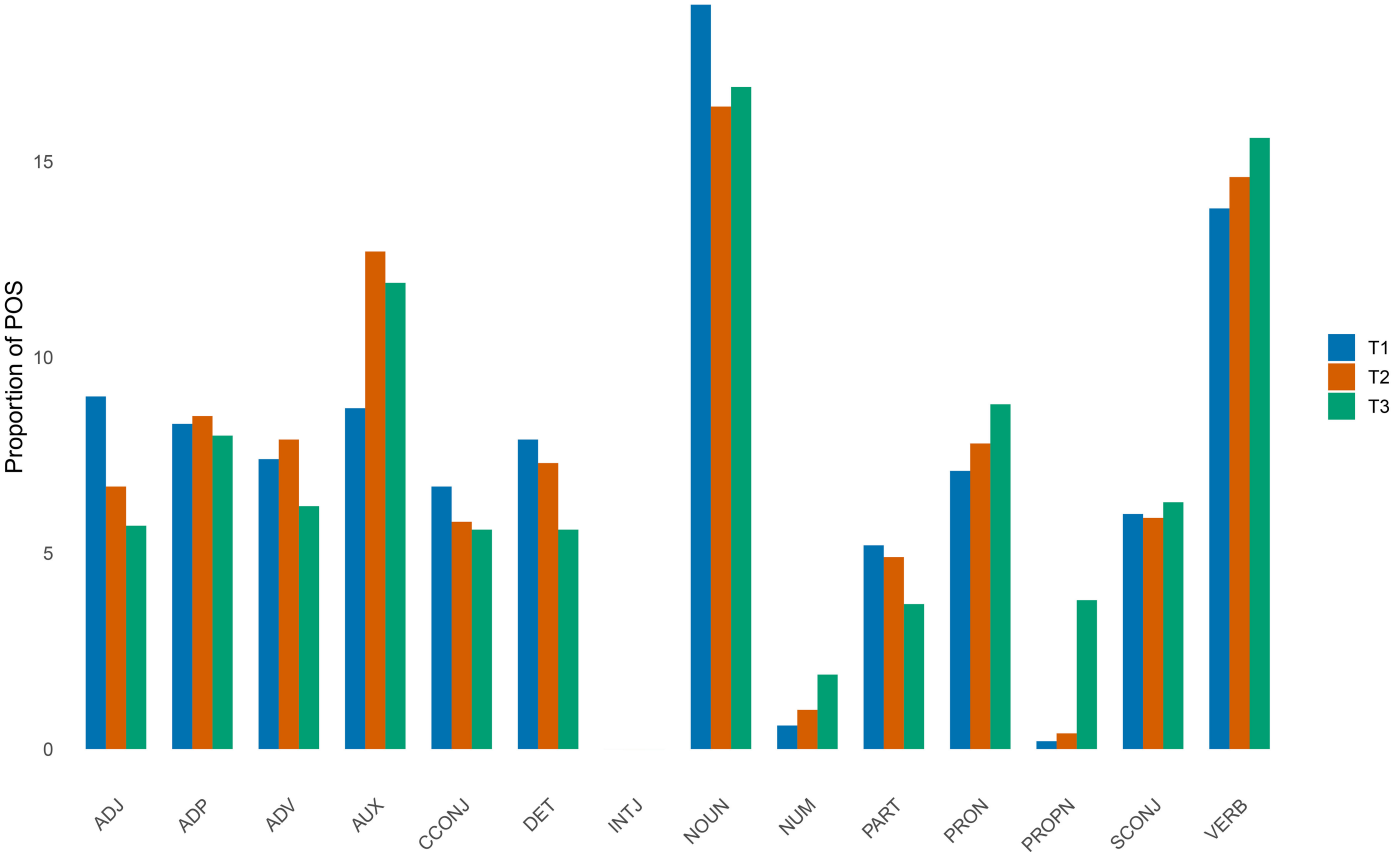
Frequencies of parts-of-speech (POS) in T1, T2, and T3.

As shown in **Figure 2**, the most notable difference lies in the use of proper nouns (PROPN), which refer to specific individuals, places, or objects. The patient uses approximately ten times more proper nouns during the manic phase (T3) than otherwise. Additionally, the use of numerals (NUM) is markedly increased in the manic phase.

Next, we assessed sentence length, defined as the average number of words per sentence, and clause density, which refers to the number of clauses per sentence. The results are presented in **Table 2**.

**Table 2:** Measures of syntactic complexity.

Measure of syntactic complexity	Text T1	Text T2	Text T3
Number of sentences	3158	5512	3815
Average number of words per sentence	11.4	11.7	12.2
SD Number of words per sentence	7.0	6.4	10.6
Average number of clauses per sentence	2.0	2.0	2.2
SD Number of clauses per sentence	1.3	1.2	1.8

The average number of words per sentence remains relatively consistent across the three texts, with T3 showing only a modest increase—7% compared to T1 and 4% compared to T2. A similar pattern is observed in the number of clauses per sentence, where T3 exhibits a 10% increase relative to both T1 and T2. However, as with lexical diversity, the manic phase is characterized by greater variability. This is reflected in the substantially higher standard deviations for both sentence length and clause density: sentence length in T3 shows a 65% increase in variability compared to T2 and a 51% increase compared to T1, while clause density exhibits a 50% increase relative to T2 and a 38% increase relative to T1. The increased variability in syntactic and lexical markers during manic states is consistent with Benjamin’s disrupted temporal integration and self-reflection. While the average sentence and clause structure remains stable, the fluctuation itself may reflect narrative instability and offers support for the clinical characterization of manic thought as both expansive and fragmented.

While the average values of standard semantic and syntactic measures show little difference between the manic and euthymic phases, the standard deviations differ notably. The summary of our multimodal analysis of Benjamin’s self-reflective texts is summarized in **Table 3**.

**Table 3:** Summary of phenomenological, narratological, and linguistic analysis of Benjamin’s psychopathology.

Dimension	Text 1: Being—Confession	Text 2: Being—Theory	Text 3: Grabbing and Absolution
Narrative Self	Experiencing self dominates; minimal reflection; outer life described with internal distance	Insight-driven self; abstract reasoning and worldview construction; fragmented epistemology	Disrupted self; minimal coherence; reflection absent; overwhelmed by factual content
Temporal Structure	Linear but emotionally flattened; focused on personal past	Nonlinear; contains existential time (infinite past, future consequences)	Disintegrated temporality; associative leaps, manic acceleration
Linguistic Profile	Moderate lexical diversity; narrative stability	Most stable; low variability; consistent formality	High variability in TTR, sentence/clause length; many proper nouns and numerals; linguistic disorganization
Phenomenological Markers	Self-objectifying emerges subtly; detachment from body and emotions begins to show	Embodied detachment thematized; philosophical synthesis of lived experience	Collapse of self-world boundary; heightened agency and spiritual symbolism
Mode of insight	Introspective but inconsistent; focused on external events	Theoretical and symbolic; high abstraction, technicist metaphors	Insight limited to event sequencing; existential overwhelm obstructs integration

### 3.5 Synthesis

In this chapter, we will attempt to synthesize the three analyses described above. We will do this in two steps. First, we will look into the integration of the form (linguistic analysis) and content (narratological analysis) of Benjamin’s self-reflections.

In phenomenological thought, the experience of one’s body is commonly divided into the body that one is [Ger. *Leib*] and the body that one has [Ger. *Körper*] (55). Earlier, we pointed out that for Benjamin, his experience of his embodiment is primarily that of *Körper*. This split, however, does not only refer to his bodily feelings. It intersects with feelings of “otherness,” which may be psychodynamically grounded in his somatic condition (*situs inversus*) and difficult upbringing.

Benjamin turned to religion, not as a deeply felt spiritual experience, but a logical system that would lead to self-understanding. For him, religion is not a specific way of being but rather a state of “being a good person” as confirmed by others. This is reflected in him frequently asking about this, usually at the end of the interviews. This process of reifying his experience into text appears to both reflect and reinforce a narrowing of his experiential field. This constant reification may be preventing him from entering dynamically and spontaneously into the shared, intersubjective space. For him, interpersonal relationships have to adhere to the rules he arrived at during his self-reflections. Thus, the lived texture of his life ossified into a series of rigid rules and a few self-defining memories that he brings up time and time again.

Access to his self-reflective texts offers clues about this process of ossification. *Grabbing and Absolution* is a text written during a manic phase. Although it was produced later than the other texts, we will, for analytical purposes, describe it first, as it comes the closest to a description of momentary, affectively charged lived experience. In the text, the self is seen as fragmented, there is no clear narrative structure. Past, present, and future are all described as if occurring simultaneously. There is a collapse of self-world boundaries reminiscent of moments of magical thinking and a sense of being in contact with a spiritual dimension. At the linguistic level, we observed high variability in TTR and sentence lengths: long descriptions of a disorganized stream of consciousness are interspersed with brief syllogism-like statements (what Benjamin would later call insights).

*Being—Confession* is a text that Benjamin wrote during a depressed state. The narrative is stable. The self gradually becomes more coherent. At the linguistic level, it exhibits modest lexical stability. The narrative progresses linearly and the affective charge is flattened. Here, we start to see a gradual reconstitution of narrative distance, which is primarily seen in Benjamin describing his life in terms of objective events that happened to him (consisting almost exclusively of self-defining memories).

Finally, *Being—Theory* is a text that was written in a euthymic state. It exhibits low lexical variability. The language is highly formal. It lacks descriptions of emotions or embodied experience. It is theoretical, abstract, and highly symbolic. Short, simple sentences describe a philosophical synthesis of his lived experience into a worldview. Benjamin employs various metaphors to articulate universal truths. Although formalistic, this texts nonetheless reflects his manic experience: his epistemology is fragmented drawing on different systems of knowledge (e.g., electrical engineering, psychology, theology) to account for his lived experience.

Throughout his attempts of (self)symbolization, Benjamin’s primary mode of existence is not one of *being-in-the-world* but of *being-in-interpretation*—a stance in which his life and is continuously externalized. His self-understanding is assembled across textual artefacts and narratives through which he constructs a self that must be translated before it can be lived. While from a normative and universalist lens of phenomenology (56, 57), this mode of being may seem altogether maladaptive, we do not wish to make that assertion. In fact, for Benjamin, his philosophical self-reflection has proven to be an essential coping mechanism. Indeed, throughout adulthood, he was able to resolve his difficulties with social cognition. His interpersonal relationships may seem odd (i.e., he primarily navigates them by relying on his technicist worldview). However, he is able to foster close ties with his inner family and friends, which provide him with psychological support and meaning in life (e.g., wanting his children to lead a good life).

The phenomenological interviews were conducted most recently. Particularly salient descriptions of his lived experience emerged from discussing interpersonal situations where he was able to veer away from his worldview. Benjamin appraises such moments of flexible, participatory sensemaking, as especially significant. Benjamin has pronounced difficulties in both engaging in and understanding such processual, intersubjective dynamics. He, at one point, remarked that over the years he had successfully “manipulated” his wife so that their domestic interactions became more in line with his wishes. He is unable to recognize that this interpretation is incompatible with them going on trips for her vacation and, at her request, attending weekly dancing lessons. This suggests a split between Benjamin’s propositional reasoning and his lived relational reality. While he interprets his influence on others as manipulative and unilateral, his actual relationships reflect reciprocity and mutual negotiation. When directly asked about these manipulations, Benjamin describes exactly the kinds of processual sensemaking described above. We interpret these moments of processual sensemaking as being insufficiently integrated into Benjamin’s worldview.

This integrative synthesis can shed light on specific clinical considerations. A psychological examination focused solely on surface traits might risk characterizing Benjamin’s personality structure as “rigid.” Such an interpretation may not sufficiently appreciate how what appears as “rigidity” is experienced functionally as a coping mechanism. Furthermore, such an analysis might misattribute his self-critical interpretations of processual sensemaking to a personality disorder. In fact, antisocial personality disorder was briefly considered in his clinical psychological DDX!

At 18-month follow-up, Benjamin reports that through the detailed multimodal investigation that we conducted, he understands himself better. He feels more emotionally stable. He learned to translate the mutual negotiation of meaning that is inherent in constructivist grounded theory (40) into daily life where he is now able to engage in difficult social situations without distancing himself when it does not conform to his rules but is able to engage in follow-up steps. Similarly, he reports that through this multimodal understanding of his experience, he was able to form a deep therapeutic alliance with the research team.

## 4 Discussion

We presented a detailed multidisciplinary case study of Benjamin, a patient with comorbid bipolar disorder and adjoined difficulties in social cognition and affectivity that are likely to be developmental in origin. A DDX of Asperger’s syndrome and schizotypal personality disorder was considered. The latter was rejected. The determination of the former could not be made since he was undiscovered until middle adulthood and developed effective coping strategies (e.g., learning to maintain eye contact). Benjamin’s stance of being-in-interpretation highlights a mode of existence shaped not by immediate immersion in the world but by continuous symbolic externalization. This mode is not merely symptomatic but existentially significant: it serves as a coping mechanism, a source of coherence, and a means of reclaiming agency.

Jaspers (17) writes that in order to understand individuals’ unique experiences of psychopathology, it is necessary to consider their lived experience, behavioral performance (e.g., on a psychological task), and productive capacities (e.g., the artworks that they make). Researchers in the field of art therapy have noted that creating art reinforces patients’ sense of self (58) primarily by leaving a tangible fact of their existence (59, 60) and distancing themselves from their often confusing altered experience (60, 61). Artistic production conferring a sense of competence is readily seen in Benjamin. He is well-read in psychology, primarily in the psychoanalytic tradition (he is particularly fond of McWilliams (62)), and he uses terms of art (e.g., referentiality, tangential stream of consciousness). Benjamin’s coping through autobiographical writings reflects the story of Wouter Kusters (63), a linguist who suffered a psychotic episode. Afterwards, he became a philosopher and posited a novel understanding of human consciousness based on his experience. Benjamin’s case may provide further avenues for how art therapy or bibliotherapy could be used to cope with traumatic past experiences.

Our multi-perspective analysis of Benjamin’s lifeworld offers us an insight into the cognitive processes wherein a worldview is constructed. Through Benjamin’s marked experience of embodiment and temporality, we can observe a lifeworld that inherently presents itself as uncertain: Benjamin is overburdened with the causes of his condition that lie somewhere in the (sometimes distant) past (e.g., intergenerational trauma, potential genetic causes of his condition, the impact of the sociocultural conditions of socialist Yugoslavia) as well as the consequences of his actions that reach far into the future. As such, Benjamin’s lifeworld is characterized by a pronounced epistemic need (24).

The close relationship between the experience of time and agency can be tied to Benjamin’s faith. It is unclear whether his experience of lived time parallels the temporality as it is understood by Christian theology or if he is simply interpreting his experience through religious teachings. In Christianity, there are two conceptions of time: *chronos* (the human time) and *kairos* (the divine time). As such, present and infinity co-exist (64). DeRoo (65) analyzes this experience in terms of eschatology (the study of the end of the world), where the lives of the faithful arc towards the final significance of the world. Similarly, Theissen (66) puts forward the notion of transparency, where in the religious experience, the everyday world becomes transparent towards some religious significance, in this case, the ultimate meaning of our actions.

Benjamin ascribes a lot of significance to unusual events in his life (e.g., moments of synchronicity), as well as the persistent reminders of his “otherness” (e.g., his *situs inversus totalis*). Benjamin does not experience *Anderssein* (67), the unspecific feelings of otherness that are characteristic of psychosis (68). He has very concrete evidence for what differentiates him from the society at large. This state of affairs is associated with what we termed being-in-interpretation, a persistent, baseline state of attempting to make sense of his life. According to Schütz (69), we need to rely on a pre-given and pre-reflective stock of knowledge to pragmatically orient ourselves in the world. For Benjamin, any received stock of knowledge is untenable due to his clinical history. He therefore feels compelled to construct an idiosyncratic worldview or what we elsewhere referred to as a personal cosmology (70).

The ongoing process of constructing a personal cosmology, however, establishes an experiential distance from his own life reflected in his texts through narrative distance and semantic variability. Such an existential detachment is commonly seen in psychiatric disorders ranging from depression (71) to psychosis (57), wherein the taken-for-granted, common-sense view of the world is lost. We thus see a cyclical dynamic in Benjamin’s lifeworld (or, what has sometimes been termed dynamic co-emergence) (72, 73). Benjamin’s feelings of alienation prompt him to construct an idiosyncratic worldview, which, in turn, reinforces his feelings of alienation.

Interestingly, increased variability in semantic coherence has also been reported in patients with psychosis (74), and is thought to reflect poor executive control over semantic selection (75). Semantic coherence is thought to reflect the associative interconnectedness of linguistic representations (76). This combined with Benjamin’s technicist self-understanding (commonly expressed in the form of specific laws that he repeats verbatim) this may suggest a difficulty in coherent processing of emotionally grounded concepts.

A useful concept to help us understand Benjamin’s experience is participatory sensemaking. This term refers to the phenomenon whereby in interpersonal interaction, the conversation is at the same time dependent and independent of both the interlocutors (77). The interaction takes on a life of its own. Participatory sensemaking is related to the notion of relational autonomy: in social interactions, meaning is intersubjectively negotiated through our mutual recognition of each other’s perspectives, rather than relying on one person’s worldview in interpersonal interaction we tend to procedurally and flexibly generate novel norms depending on the specific context, often without referring to pre-existing patterns. Participatory sensemaking includes a dynamic coupling of both the self and the other (78). Benjamin’s primary difficulties with social cognition and his secondary idiosyncratic worldview preclude such an interaction, leading to continuous emotional turmoil.

This case provides an example of how some intellectual endeavors, even those that may initially seem eccentric, overly intellectual, or even incoherent, can serve a existential, meaning making functions and promote self-constitution. Ultimately, Benjamin’s case invites a reconsideration of what it means to “understand” a psychiatric patient in terms of how a person attempts to live, interpret, and make sense of being. Benjamin’s self-reflections and his worldview are deeply meaningful to him. Labeling these as “maladaptive cognitive schemata” may be reductionist. We advise clinicians to reflect on how such processes could serve important meaning making functions for the individual.

## 5 Conclusion

We presented an interdisciplinary (phenomenological, clinical psychological, narratological and linguistic) case study of Benjamin, a patient with a complex clinical picture. Benjamin’s lifeworld is radically different from others in his immediate social environment. This has prompted him to reflect not only on his experience but also on the world as a whole. He has written five book-long texts in which he constructed a worldview based on his experience. We opened a discussion on how his lived experience, personal cosmology (his pre-reflective experience of what the world is and how it works), and pervasive self-narrative constitute a novel worldview that, while adaptive for understanding his psychopathology, maintains his feelings of alienation and thereby opens him up to future emotional distress.

The present study demonstrates how multidisciplinary analysis of multiple forms of qualitative material can be used to better understand patients’ attitudes towards their disorder, in particular in complex clinical pictures emerging from multiple comorbidities and lifelong attempts to cope with distress. It would be of interest to see how in the future, such a methodology could be streamlined and applied to larger samples such that causal relationships between patterns of sensemaking and psychopathology could be established.

## Data Availability

The raw data supporting the conclusions of this article will be made available by the authors, without undue reservation.

## Footnotes

1 Throughout the text, we attempt to connect different meaningful connections as reported by Benjamin, observed by the researchers, and indicated by the analysis of multimodal data. It is imperative for the reader to keep in mind that our assertions here are not causal. We are following what Jaspers (17) referred to as *genetic phenomenology:* the identification of typical descriptive patterns. If at any point, causal dynamics are implied, this should be read as an artefact of language.

2 Inheritance and inheritance laws exhibit culturally specific dynamics in post-socialist, particularly Slavic societies. These were traditionally patriarchal with patrilineal inheritance. With the end of socialism, property laws changed leading to a renegotiation of social relationships. Family members litigating each other over inheritance is thus a common phenomenon.

3 He is nonetheless eclectic in his choices of organizing frameworks. He is notably not interested in neuroscience. A colleague once explained to him the relationship between the fear response and the amygdala, which he consistently mispronounces as *amalgala*.

4 The actual phrase he used was *neperspektivno*, “lacking perspective.” It is difficult to translate the exact Slovenian usage of this term into English. It broadly means “having a promising future.” A choice of study or a romantic partner is commonly described as *perspektivno*.

5 Quotes from Benjamin's texts are written out in italics.

6 The notion of *un-understandable* comes from Jaspers (17). In *General Psychopathology*, he differentiates between scientific explanation [Ger. *Erklärung*] of the mind (as revealed, for example, with an experiment) and our own understanding [Ger. *Verständnis*]. In constructing understanding, we necessarily have to create meaningful connections between our own intrapsychic phenomena as they are revealed to us. This, however, by necessity of the limitations of our insight, confronts us with un-understandable [Ger. *Unverständliche*].
